# Tilivalline- and Tilimycin-Independent Effects of *Klebsiella oxytoca* on Tight Junction-Mediated Intestinal Barrier Impairment

**DOI:** 10.3390/ijms20225595

**Published:** 2019-11-08

**Authors:** Nina A. Hering, Anja Fromm, Roland Bücker, Gregor Gorkiewicz, Ellen Zechner, Christoph Högenauer, Michael Fromm, Jörg-Dieter Schulzke, Hanno Troeger

**Affiliations:** 1 Medical Department of General, Visceral and Vascular Surgery, Charité – Universitätsmedizin Berlin, 12203 Berlin, Germany; 2 Institute of Clinical Physiology/Nutritional Medicine, Medical Department, Division of Gastroenterology, Infectiology and Rheumatology, Charité – Universitätsmedizin Berlin, 12203 Berlin, Germany; anja.fromm@charite.de (A.F.); roland-felix.buecker@charite.de (R.B.); michael.fromm@charite.de (M.F.); joerg.schulzke@charite.de (J.-D.S.); 3 Institute of Pathology, Medical University of Graz, A-8036 Graz, Austria; gregor.gorkiewicz@medunigraz.at; 4 BioTechMed-Graz, Institute of Molecular Biosciences, University of Graz, A-8010 Graz, Austria; ellen.zechner@uni-graz.at; 5 Division of Gastroenterology and Hepatology, Medical University of Graz, A-8036 Graz, Austria; christoph.hoegenauer@medunigraz.at; 6 Medical Department, Division of Gastroenterology, Infectiology and Rheumatology, Charité – Universitätsmedizin Berlin, 12203 Berlin, Germany; hanno.troeger@charite.de

**Keywords:** antibiotic-associated hemorrhagic colitis, tight junction, claudin, apoptosis, *Klebsiella oxytoca*

## Abstract

*Klebsiella oxytoca* causes antibiotic-associated hemorrhagic colitis and diarrhea. This was attributed largely to its secreted cytotoxins tilivalline and tilimycin, inductors of epithelial apoptosis. To study whether *Klebsiella oxytoca* exerts further barrier effects, T84 monolayers were challenged with bacterial supernatants derived from tilivalline/tilimycin-producing AHC6 or its isogeneic tilivalline/tilimycin-deficient strain Mut-89. Both preparations decreased transepithelial resistance, enhanced fluorescein and FITC-dextran-4kDa permeabilities, and reduced expression of barrier-forming tight junction proteins claudin-5 and -8. Laser scanning microscopy indicated redistribution of both claudins off the tight junction region in T84 monolayers as well as in colon crypts of mice infected with AHC6 or Mut-89, indicating that these effects are tilivalline/tilimycin-independent. Furthermore, claudin-1 was affected, but only in a tilivalline/tilimycin-dependent manner. In conclusion, *Klebsiella oxytoca* induced intestinal barrier impairment by two mechanisms: the tilivalline/tilimycin-dependent one, acting by increasing cellular apoptosis and a tilivalline/tilimycin-independent one, acting by weakening the paracellular pathway through the tight junction proteins claudin-5 and -8.

## 1. Introduction

A vast number of microorganisms colonize the intestinal mucosa, forming a complex ecosystem. The importance of this diverse microbiota for intestinal health and function becomes evident once the balance of this ecosystem is disturbed. This can occur by overgrowth of a single species after antibiotic medication, as it is well-known from the toxinogenic strains of *Clostridium difficile* causing antibiotic-associated diarrhea and pseudomembranous colitis [1]. Within the last years, *Klebsiella oxytoca* (*K. oxytoca*) was recognized to cause antibiotic-associated hemorrhagic colitis (AAHC), a distinct form of antibiotic-associated colitis, accompanied by bloody diarrhea and abdominal cramps [2]. *K. oxytoca* is a Gram-negative bacterium that is ubiquitous in the environment, but also colonizes the healthy intestinal mucosa [2,3,4] and is associated with nosocomial infections [5]. Most clinical isolates are resistant to amino- and carboxypenicillins [6], favoring selective intestinal overgrowth of *K. oxytoca* in antibiotic-treated patients.

*K. oxytoca* secretes cytotoxic compounds into the external milieu in vitro and in the host intestine [7,8,9,10]. Schneditz and colleagues identified a cluster of cytotoxin synthesis genes that is shared by toxin-positive *K. oxytoca* strains. They showed the pentacyclic pyrrolobenzodiazepine tilivalline to be responsible for inducing epithelial apoptosis in Hep-2 cells in vitro, and that the biosynthetic gene cluster was required for disease in a mouse model of AAHC. Subsequent studies showed that the gene cluster produces a second pyrrolobenzodiazepine toxin, tilimycin, which also induces apoptosis in epithelial cells and contributes to the development of colitis [9,10,11,12]. Knock-out of the *npsB* gene, encoding a nonribosomal peptide synthetase within the cluster, exhibited a toxin-negative phenotype and was incapable of inducing apoptosis [8].

The “leaky gut concept” is defined by an impaired paracellular barrier, resulting in increased flux across that barrier [13]. Consistent with that concept, stimulated epithelial apoptosis was demonstrated to have a direct leak effect [14]. Tilivalline causes a decrease in transepithelial resistance in epithelial T84 monolayers, indicating an impaired barrier function. Interestingly, this tilivalline-induced decrease could be abolished when apoptosis was inhibited pharmacologically [8]. Epithelial apoptosis is also a feature of barrier dysfunction induced by other bacterial enteropathogens, for example, *Campylobacter spp.* [15,16] or *Arcobacter buzleri* [17]. Other studies showed epithelial apoptosis can induce cleavage of cell adhesion molecules [18,19,20]. 

The epithelial tight junction forms a sealing structure between the lateral cell membranes of adjacent epithelial cells and is built by different types of transmembranal proteins. The two types capable of barrier formation are the claudin family, with 27 members in mammals [21], and the family of TJ-associated MARVEL proteins (MAL and related proteins for vesicle trafficking and membrane link), comprising occludin, tricellulin, and marvel-D3 [22]. Extracellular loops of these proteins interact with those from the neighboring cells, and by this, build up the paracellular barrier. However, some of the claudins form paracellular channels for small cations, anions, or water. Under pathological conditions, upregulation of channel-forming claudins or downregulation of barrier-forming claudins lead to barrier dysfunction [23]. Because of this key function for intestinal barrier function, the present study aimed to clarify how *K. oxytoca* affects tight junction protein function.

## 2. Results

**Figure 1:**
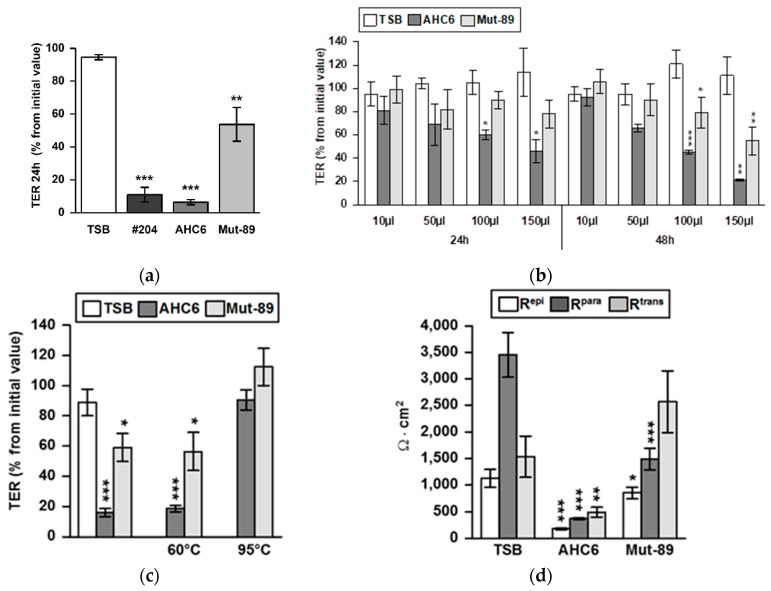
Tilivalline/tilimycin-dependent and -independent effects on transepithelial resistance. (**a**) T84 monolayers were infected with vital tilivalline/tilimycin-producing *K. oxytoca* strains #204 and AHC6 or the AHC6 isogenic mutant strain Mut-89. TER (transepithelial resistance) levels were measured at 24 h after infection and were compared with initial values (set 100%) and untreated controls (*n* = 5–7; ** *p* > 0.01; *** *p* < 0.001 versus control). (**b**) Dose- and time-dependent TER effects of preparations of bacterial culture supernatants from *K. oxytoca* strains AHC6 and from tilivalline/tilimycin knock-out Mut-89 were assessed on T84 monolayers. Controls were treated with equal amounts of TSB culture medium (*n* = 4–5; * *p* < 0.5; ** *p* < 0.01; *** *p* < 0.001 versus TSB). (**c**) Supernatants of ACH6 or Mut-89 were heated to 95 or 60 °C prior to T84 challenge and TER measurements (*n* = 5–10; *p* < 0.05, *p* < 0.001 versus TSB). (**d**) Discrimination of paracellular (R^para^) and transcellular resistance (R^trans^) by two-path impedance spectroscopy was performed 48 h after challenging cellular monolayers with supernatant of AHC6 or Mut-89 (*n* = 4–13; ** *p* < 0.01; *** *p* < 0.001 versus TSB control).

After infection of T84 monolayers with vital tilivalline/tilimycin-producing *K. oxytoca* strains AHC6 or #204, we observed a strong drop in transepithelial resistance (TER) within 24 h (Figure 1a). For comparison, we infected the monolayers with the toxin-negative AHC6 mutant strain Mut-89, which is incapable of producing tilivalline and tilimycin. Interestingly, the Mut-89 strain decreased TER as well. To assess the role of tilivalline and tilimycin, we prepared supernatants of broth cultures of the cytotoxin-producing strain AHC6 and the mutant strain Mut-89. Different doses of supernatant preparations (10, 50, 100, and 150 µL) of AHC6 or Mut-89 were assessed after 24 and 48 h. As expected, the toxin-positive strain reduced TER in T84 monolayers in a dose- and time-dependent manner. However, we also observed a reduction in TER by the mutant strain, although AHC6 was more effective than Mut-89. Since the strongest effect was observed with 150 µL at 48 h (Figure 1b), this setup was used for all following cell culture experiments. To further differentiate the nature of the supernatants derived from – AHC6 and Mut89, thermal stability was tested by heating. TER effects of supernatants of AHC6 and Mut-89 were abolished by heat treatment at 95 °C, but not at 60 °C. Supernatants treated at 60 °C were as effective as native preparations (Figure 1c). TER (or epithelial resistance, R^epi^) consists of a paracellular (R^para^) and a transcellular (R^trans^) component. While the effects of AHC6 supernatant on R^epi^ were due to a severe reduction of both R^para^ and R^trans^, the R^epi^ drop caused by supernatant of the mutant strain Mut-89 was caused solely by a decrease in R^para^ (Figure 1d).

These findings were in accordance with permeability changes of the paracellular marker molecules fluorescein (332 Da) and FITC-dextran-4000 (FD4, 4 kDa). Here, AHC6 enhanced the permeability for fluorescein about 14-fold and for FD4 about 4-fold compared with TSB-treated monolayers (Table 1). Preparations of Mut-89 also increased permeability, but the effect was less pronounced than with AHC6. Fluorescein permeability was increased about 2-fold and FD4 permeability about 1.6-fold versus TSB control (Table 1).

**Figure 2:**
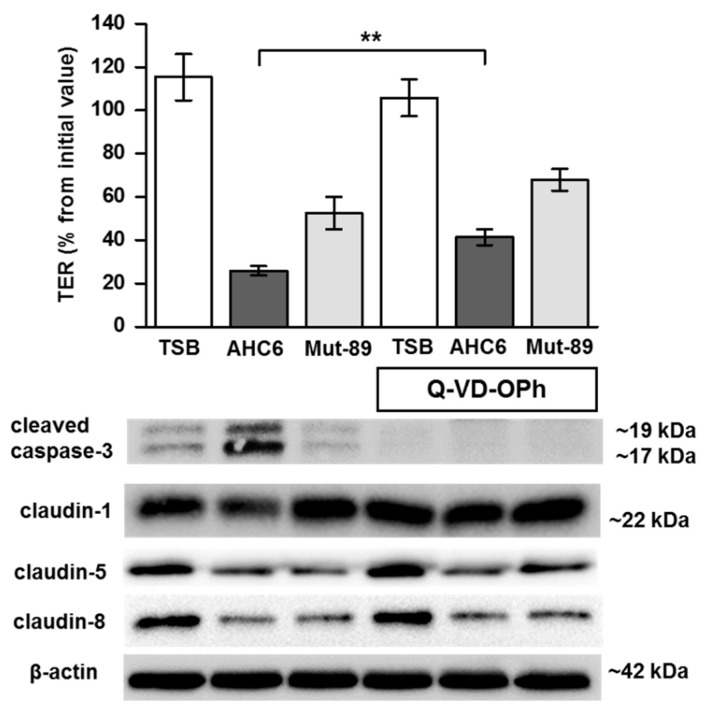
Impact of apoptosis inhibition on transepithelial resistance and tight junction protein expression. T84 monolayers were pretreated with apoptosis inhibitor Q-VD-OPh before challenging with supernatants from AHC6 or Mut-89. Control monolayers were treated with TSB. TER measurement was performed over 48 h (*n* = 7; ** *p* < 0.01), followed by analysis of protein expression. Blocking of Caspase-3 cleavage served as positive control for apoptosis inhibition by the pan-caspase inhibitor Q-VD-OPh (bars on the right). A representative Western blot shows tilivalline/tilimycin-dependent and -independent tight junction changes (*n* = 5–6).

To assess the impact of tilivalline/tilimycin-dependent epithelial apoptosis on TER and tight junction changes, T84 monolayers were treated with Q-VD-OPh, a potent inhibitor of caspase-3 cleavage, prior to challenging with *K. oxytoca* supernatants (Figure 2). TER in monolayers treated with Q-VD-OPh and AHC6 supernatant was not significantly different from that challenged with Mut-89 supernatant (without Q-VD-OPh), but was lower than in TSB controls, pointing to an apoptosis-independent mechanism. Interestingly, Q-VD-OPh treatment abolished the AHC6-induced TER decrease only by about 15.5% but inhibited caspase-3 cleavage completely (Figure 2).

We next examined the expression profile of distinct barrier-relevant tight junction proteins by Western blotting and subsequent densitometry (Table 2). Challenging for 48 h with AHC6 or Mut-89 supernatants reduced protein levels of claudin-5 and -8 to a similar extent, when compared with TSB controls. In addition, supernatant from AHC6, but not from Mut-89, reduced claudin-1 protein level, which was prevented by pretreatment with Q-VD-OPh. The expression of claudin-2, -4, or tricellulin was neither affected by AHC6 nor by Mut-89 supernatants (Table 2). In Q-VD-OPh-treated monolayers, claudin-1 protein expression did not differ from that in TSB controls, while protein levels of claudin-5 and -8 remained significantly different (Table 2, and signal intensity in Figure 2).

**Figure 3:**
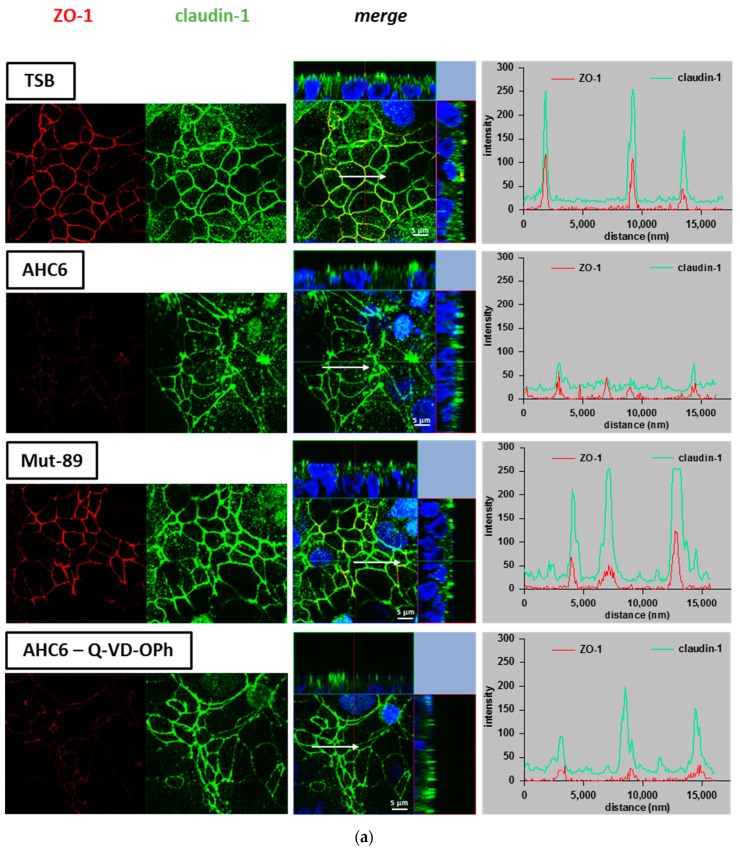

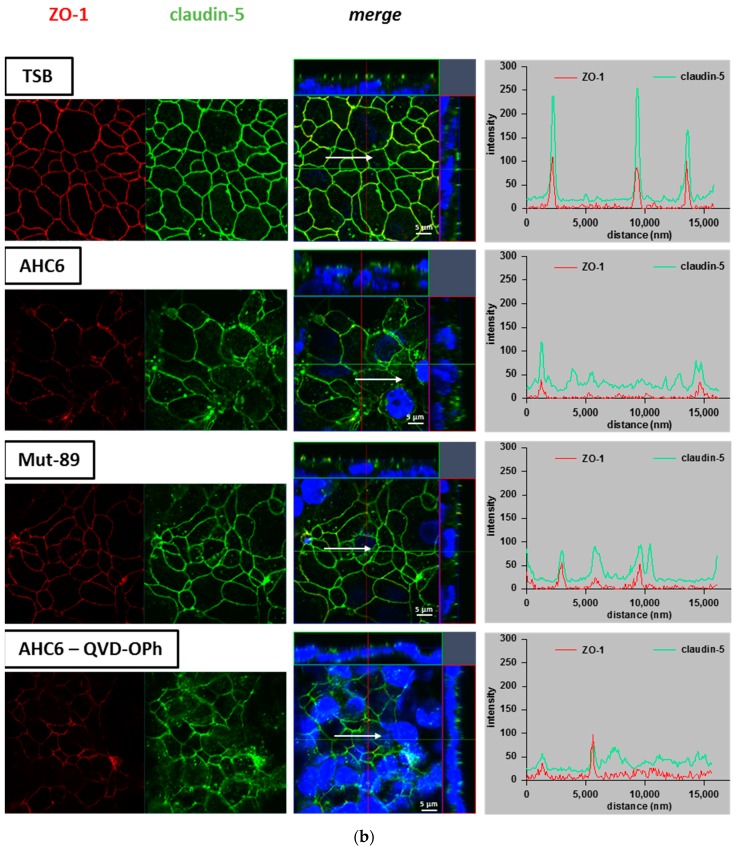

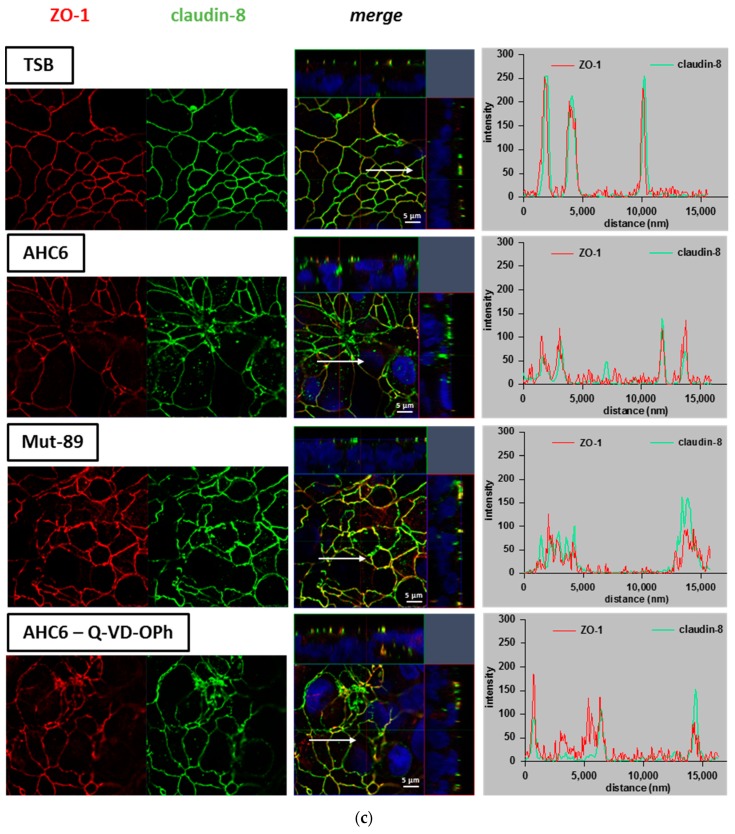
Redistribution of tight junction proteins in T84 monolayers. T84 monolayers were immunostained after 48 h of challenging with supernatants from AHC6, with or without previous apoptosis inhibition (Q-VD-OPh) or supernatants from Mut-89. TSB-treated monolayers served as control. Immunofluorescence staining of (**a**) claudin-1, (**b**) claudin-5, and **c)** claudin-8 (green) were analyzed by confocal laser scanning microscopy (*n* = 3 each). ZO-1 (red) served as tight junction marker. Distribution of tight junction proteins was considered by z-stack imaging. Intensity–distance plots show the signal intensity and merging of ZO-1 and claudin signals in representative areas. Arrows were located using Zeiss Zen software and mark the range and direction of the intensity–distance plot. Nuclei were DAPI-stained (4′,6-Diamidin-2-phenylindol; blue).

Beside protein expression, the proper functional localization of tight junction proteins was investigated by immunostaining and subsequent laser scanning microscopy (LSM) on T84 monolayers. The zonula occludens protein 1 (ZO-1) served as a tight junction marker. We monitored the localization and signal intensity of claudin-1, -5 and -8 by z-stack imaging and intensity–distance plots of representative areas. Effects of AHC6 supernatants were investigated without and with Q-VD-OPh apoptosis inhibition and were compared with effects of Mut-89 supernatants and TSB-treated control. Monolayers challenged with AHC6 supernatant showed claudin-1 to be redistributed out of the tight junction domain of the cells, especially within areas of apoptotic foci (Figure 3a, second row). In contrast, intensity–distance plots of control monolayers showed a clear merging of red (ZO-1) and green (claudin-1) peaks with high intensities (amplitudes) at regions of cell contact (tight junctions), and signal intensity in intracellular regions was very low (background intensity). In AHC6-treated monolayers, the intensities of claudin-1 and ZO-1 signals were reduced and more diffuse (Figure 3a, second row). While ZO-1 localization seemed unaffected, intensity–distance plots showed irregular and flattened curves, pointing to reduced protein expression and redistribution of claudin-1 off the tight junction domain of the cell. Claudin-1 changes in signal intensity and localization were relieved by Q-VD-OPh apoptosis inhibition, while ZO-1 intensity reduction was unaffected by Q-VD-OPh (Figure 3a, fourth row). Monolayers treated with Mut-89 supernatant looked similar to those of the TSB-controls (Figure 3a, third row).

In contrast to claudin-1, signal intensity and focal localization of claudin-5 (Figure 3b) and claudin-8 (Figure 3c) were affected by both AHC6 and Mut-89 supernatants. Overall, signal intensity was reduced in challenged monolayers compared with control monolayers (Figure 3b,c, second and third rows). The intensity–distance plots of controls showed single matching peaks of red and green curves and only slight background signal. However, plots of monolayers treated with *K. oxytoca* supernatants revealed smaller amplitudes of green (claudin-5 or -8) and red (ZO-1) curves. Focally enhanced intracellular claudin signals were indicated by irregular curves, due to enhanced background or additional peaks in intracellular regions. Q-VD-OPh treatment did not prevent reduction of claudin-5 and -8 and also single foci of delocalization were still evident (Figure 3b and c, fourth and fifth rows). Compared with its respective controls, the signal intensity of the tight junction anchor ZO-1 was diminished in all AHC6- or Mut-89-challenged monolayers (Figure 3a–c).

**Figure 4:**
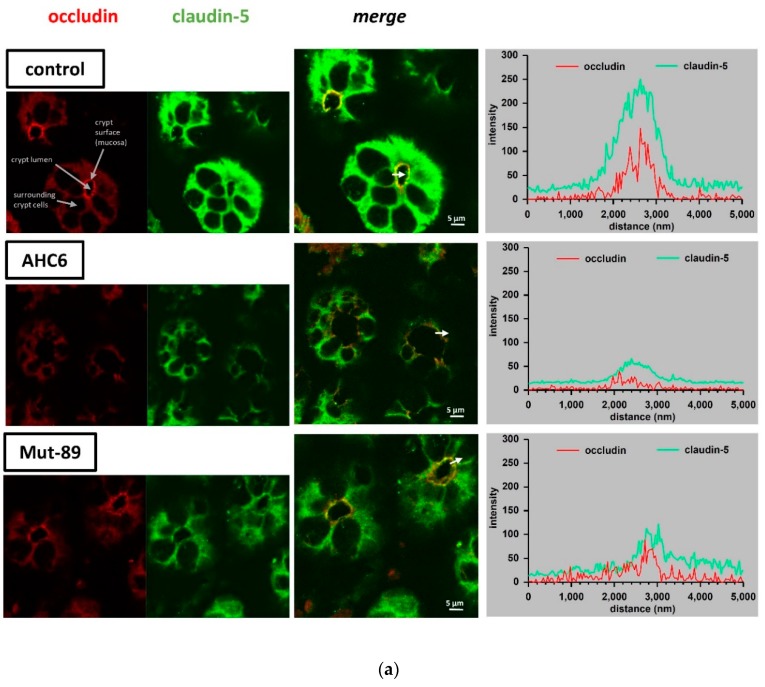

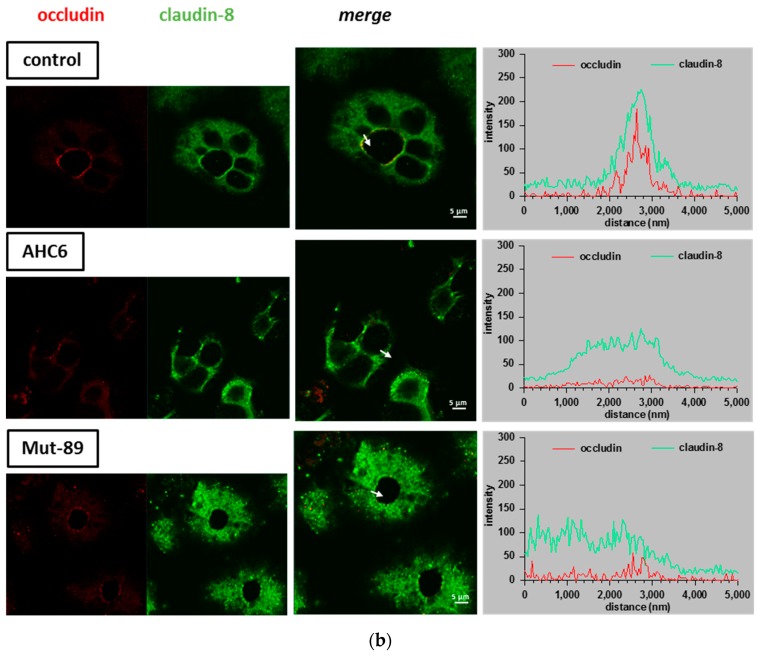
**Immunofluorescence staining of claudins in colon crypts from an AAHC** (antibiotic-associated hemorrhagic colitis) **mouse model.** Representative micrographs show immunostaining of occludin (red) and (**a**) claudin-5 or (**b**) claudin-8 (green) in mouse colon crypts of control, AHC6-infected, and Mut-89-infected mice (*n* = 4 each group; claudin-5 and -8). Signal intensity and localization of representative areas are shown in intensity–distance plots. Arrows were located using Zeiss Zen software and mark the range and direction of the intensity–distance plot.

These findings were corroborated by immunostaining of claudin-5 and -8 on colon samples, derived from a mouse model of AAHC (Figure 4). Occludin was used as a tight junction marker, and tight junction staining was analyzed in colon crypts of untreated controls and AHC6- or Mut-89-infected mice. In comparison with controls, signal intensities of claudin-5 (Figure 4a), claudin-8 (Figure 4b), and occludin (Figure 4a,b) were reduced in AHC6- and Mut-89-infected mice. Intensity–distance plots of representative areas showed reduced amplitudes of green (claudin) and red (occludin) curves. In case of claudin-8, signals were more diffuse in infected animals, and intensity–distance plots displayed a wide-ranging and flat-topped curve (Figure 4b).

## 3. Discussion

The pathomechanisms of *K. oxytoca*-induced antibiotic-associated hemorrhagic colitis are not completely understood. Recently, the cytotoxins tilivalline and tilimycin were identified and shown to cause epithelial apoptosis [8,9,10]. However, so far it was not clear if epithelial apoptosis was the only mechanism of *K. oxytoca*-caused barrier perturbation. Within the present study, we showed that *K. oxytoca*-induced barrier impairment involves not only epithelial apoptosis, but also tilivalline/tilimycin-dependent and -independent changes to the expression and localization of distinct tight junction proteins.

As expected, vital tilivalline/tilimycin-producing *K. oxytoca* isolates AHC6, and #204 induced a severe TER drop in T84 colon epithelial monolayers. Barrier perturbation in the host intestine was so far attributed to the cytotoxins tilivalline and tilimycin [8,10]. However, our study showed that vital bacteria and a cell-free preparation of bacterial culture supernatant of the mutant strain Mut-89, which is incapable of producing tilivalline/tilimycin, was also able to induce barrier dysfunction in T84 monolayers, indicated by a TER decrease and an increase in permeability for fluorescein and FD4. The biosynthesis of *K. oxytoca-*secreted compounds tilivalline/tilimycin and also culdesacin are dependent on the nonribosomal peptide synthetase operon including *npsB* [11]. Therefore, they cannot be responsible for the barrier defects caused by Mut-89, which is based on a *npsB* knock-out [8]. In a study on oral epithelial wound recovery, De Ryck and colleagues already pointed out that *K. oxytoca* must secret other effector molecules beyond tilivalline and tilimycin. They hypothesized that *K. oxytoca*-produced quorum-sensing molecules might contribute to its inhibitory impact on wound healing [24]. Former studies showed the toxicity of culture supernatant of *K. oxytoca* derived from patients with AAHC was not inactivated by 60 °C treatment, but was sensitive to 100 °C [25]. Interestingly, Mut89 supernatant preparations were also stable at 60 °C, equivalent to the wild type. Although, the particular nature of the virulence factor affecting claudin-5 and -8 remains elusive, the present study revealed a distinct pathomechanism beyond tilivalline/tilimycin-induced apoptosis.

While the AHC6 supernatant preparation reduced R^trans^ and R^para^, preparations of the tilivalline/tilimycin knock-out strain affected only R^para^, which reflects tight junction integrity. In fact, we found *K. oxytoca*-induced tight junction changes in the T84 cell culture model as well as in colon tissue derived from mice of an AAHC model. The reduction of claudin-1 and its proper localization within the tight junction turned out to be functionally dependent on the cytotoxins tilivalline and tilimycin, as the supernatant of Mut-89 did not induce any changes to this tight junction protein in vitro. In a very recent study that predominantly examined the impact of microbial balance in the gut on the microbiota–gut–brain axis, treatment of mice with ampicillin induced an imbalance of gut microbiota, with a clear prevalence of *K. oxytoca*. Interestingly, the treatment with ampicillin or with *K. oxytoca* itself suppressed claudin-1 in the colon [26]. This observation fits to our findings of a reduced claudin-1 level. Claudin-5 and -8 are barrier-forming tight junction proteins [27]. Perturbation of these proteins can be associated with barrier loss, as already found in other studies. In a mouse model of *Yersinia enterocolitca* infection, barrier defects involved a reduced expression of claudin-8 and redistribution of claudin-5 [28]. Claudin-5 and -8 are also reduced and redistributed in Crohn’s disease, which is characterized by intestinal inflammation and diarrhea [29]. Within the present study, the reduction and redistribution of these proteins occurred independently from tilivalline/tilimycin in vitro and in vivo. The observations from the animal model of AAHC supported the findings from our T84 cell cultures. Analyses of crypts of AHC6- and Mut-89-infected mice revealed that claudin-5 and -8 as well as occludin were reduced and distributed off the tight junction. In case of claudin-8, a redistribution into subapical cytoplasmatic compartments might be assumed from Figure 4b. The tight junction defects caused by *K. oxytoca* are very similar to effects observed with other enterobacterial pathogens. For example, the pore-forming toxin aerolysin, from *Aeromonas hydrophila* induces tight junction protein redistribution [30]. The *Clostridium perfringens* enterotoxin uses claudin-3, -4, and -7 as receptors to damage the epithelial cells [31]. Samples of the mucosa obtained from patients with *Camplylocbacter jejuni* infection revealed reduced expression of claudin-3, -4, -5, and -8 and redistribution of claudin-1, -5, and -8 off the tight junction [16].

Interestingly, occludin and the scaffold protein ZO-1 that serves as a tight junction anchor were also reduced by *K. oxytoca*. Both molecules have diverse regulatory functions, especially for Rho-kinase-dependent actin remodeling [32] or myosin light chain kinase-dependent trafficking [33]. ZO-1 and occludin interactions play a crucial role for proper epithelial cell polarization, as ZO-1 knock-down is associated with delayed occludin recruitment and tight junction assembly [34]. It is tempting to speculate that *K. oxytoca* causes epithelial cell depolarization similar to enteropathogenic *E. coli* [35]. In a very recent study, *K. oxytoca* was found to be increased and associated with intestinal dysfunction in cachectic mice. The authors pointed out that these effects were independent from tilivalline/tilimycin, as the *K. oxytoca* isolated from mouse feces revealed no cytotoxic activity. Here the mRNA level of occludin and immune relevant barrier factors were reduced [36]. Thus, the loss of ZO-1 or occludin might be also relevant for the barrier destabilization in *K. oxytoca* infection.

**Figure 5:**
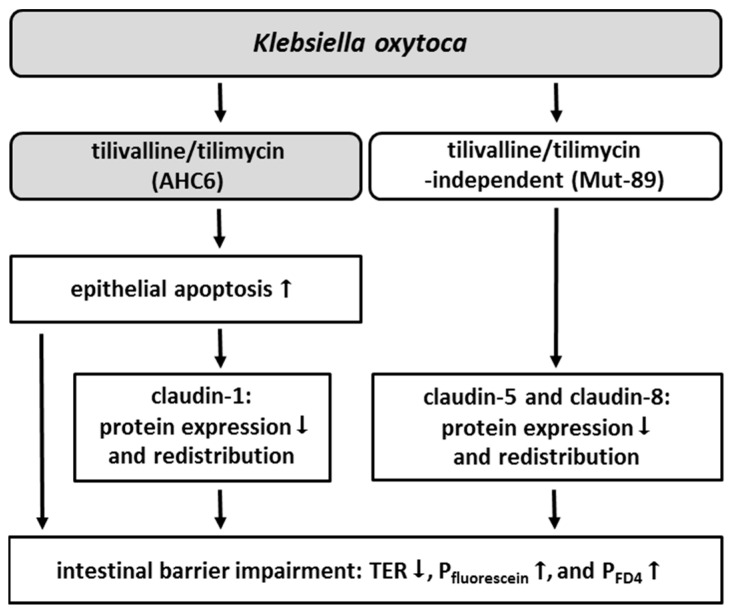
Overview of tilivalline/tilimycin-dependent and -independent effects in *K. oxytoca*-induced barrier impairment. *K. oxytoca* causes barrier impairment via two different pathomechanisms. On the one hand, the cytotoxins tilivalline and tilimycin (AHC6) enhance epithelial apoptosis, which is additionally linked to a reduction and redistribution of the tight junction protein claudin-1. On the other hand, the sealing claudins 5 and 8 are reduced and redistributed independently from tilivalline/tilimycin (Mut-89). In consequence, barrier function is impaired, indicated by reduced TER and increased permeability to fluorescein (322 Da) and FD4 (4 kDa).

In conclusion, *K. oxytoca*-induced intestinal barrier impairment involves two pathomechanisms acting in parallel. One is known to act via tilivalline/tilimycin and causes a barrier defect by increased apoptosis. As an additional indirect effect, apoptosis appears to downregulate and to delocalize claudin-1. As a further mechanism, *K. oxytoca* causes via tilivalline/tilimycin-independent action a direct weakening of the tight junction barrier by downregulation and redistribution of claudin-5 and -8 (summarized in Figure 5). This might be clinically relevant as toxin-negative *K. oxytoca* strains are commonly also found in patients with diarrhea or colitis other than AAHC [7,37].

## 4. Material and Methods

### 4.1. Cell Culture and Challenge

The epithelial colon carcinoma cell line T84 was cultured as published before [38]. Cells were grown on permeable Millicell PCF filters (0.6 cm^2^ effective area; 3 µm pores, Millicell PCF, Millipore, Schwalbach, Germany) to form polarized and tight monolayers (range 1150–2020 Ω·cm^2^). For experiments, confluent monolayers were shifted to serum-free medium, containing 100 µg/mL gentamicin in case of challenge with bacterial culture supernatants.

*Klebsiella oxytoca* strains #204 (AAHC wild type, tox^+^) [37], AHC6 (AAHC wild type, tox^+^), and Mut89 (Kan^R^; *npsB*::*Tn5*, tox^-^) [8] were cultured in Tryptic Soy Broth (TSB, Merck, KGaA, Darmstadt, Germany) overnight at 37 °C. For infection, overnight cultures were diluted (1:8) and grown for an additional 2 h. T84 monolayers were infected with vital bacteria (multiplicity of infection 10) from the apical side. To avoid bacterial overgrowth, 100 µg/mL gentamicin was added 2.5 h after infection. Bacterial culture supernatant was prepared as described earlier by Joaining and colleagues [7]. In short, 30 mL of TSB was inoculated with a single bacterial colony, and cultures were grown under gentle agitation (180 rpm) for 14–16 h at 37 °C. At an optical density at 600 nm of 4 to 6, the cultures were centrifuged (5000 rpm, 4 °C, 20 min) and supernatants were filtered through 0.2 µm cellulose acetate filters (Millipore). Monolayers were challenged with 150 µL bacterial culture filtrate or TSB for control from the apical side. To inhibit epithelial apoptosis, 10 µM of Q-VD-OPh, non-O-methylated (Merck) was added to both sides 3 h before challenge [39].

### 4.2. TER Measurement

Transepithelial electrical resistance was measured with chopstick electrodes at 37 °C, as published earlier [40]. Initial TER values were assessed before addition of bacterial supernatant or TSB. TER is given in percent of the initial value.

### 4.3. Permeability Measurements

Permeability of 0.1 mM fluorescein (332 Da, Sigma-Aldrich, Schnelldorf, Germany) or 0.4 mM dialyzed fluorescein-iso-thio-cyanate-dextran-4000 (FD4; TdB Consultancy, Uppsala, Sweden) was assessed by flux measurements in Ussing chambers under voltage-clamp conditions. The bathing solution contained in mM: 140 Na^+^, 123.8 Cl^–^, 5.4 K^+^, 1.2 Ca^2+^, 1.2 Mg^2+^, 2.4 HPO_4_^2–^, 0.6 H_2_PO_4_^–^, 21 HCO_3_^–^, 10 D(+)-glucose. The marker molecule was added to the apical side of the monolayer, and samples were collected from the basolateral side at defined time points. Fluorescence was measured in a spectrofluorimeter (Infinite M200, Tecan, Männedorf, Austria) at 525 nm, and permeability *p* (cm·s^–1^) was calculated from the ratio of flux J (mol·h^–1^·cm^–2^) over concentration Δc (mol/L) of the marker molecule in the Ussing chamber: *p* = J/Δc.

### 4.4. Measurement of Para- and Transcellular Resistance

Differentiation of paracellular (R^para^) and transcellular (R^trans^) contributions to the overall epithelial resistance (R^epi^, TER) was performed as described earlier [39,41]. Briefly, cellular T84 monolayers were mounted in Ussing-type chambers, and transepithelial conductance (G^epi^) and fluxes of the paracellular marker fluorescein were measured under voltage-clamp conditions. Changes in R^para^ were forced by chelating extracellular Ca^2+^ using ethylene glycol tetra acetic acid. Calculation was done from conductivity and fluxes before and after Ca^2+^ removal.

### 4.5. Western Blot

Proteins were extracted from epithelial T84 monolayers with ice-cold lysis buffer (in mM: 100 imidazole, 100 KCl, 300 sucrose, 2 MgCl_2_, 10 EGTA, 1 NaF, 1 NaVO_3_, 1 Na_2_MO_4_, 0.2% Triton X-100, and complete protease inhibitor cocktail (Roche, Mannheim, Germany)). For analysis of caspase-3 activation, cell lysis was performed as described earlier [39]. Twenty–thirty micrograms of protein was separated by SDS-polyacrylamid gel electrophoreses and blotted on polyvinylidene difluoride (PVDF)-membranes. For immunodetection, the following antibodies were used: anti-claudin-1, -2, -4, -5, and -8, anti-tricellulin (Thermo Fisher Scientific, Bremen, Germany), anti-cleaved Caspase-3 (Cell Signaling Technology, Denvers; USA), and anti-β-actin (Sigma-Aldrich). Bound antibodies were detected with peroxidase-conjugated goat anti-rabbit IgG or goat anti-mouse IgG antibodies using the chemiluminescence substrate Lumi-Light^PLUS^ (Roche) and the chemiluminescence detection system FX7 (Vilber Lourmat, Eberhardzell, Germany). Densitometry was performed with AIDA quantification software (Raytest, Straubenhardt, Germany). All values were normalized to β-actin.

### 4.6. Immunofluorescence Staining and Confocal Laser scanning Microscopy

T84 monolayers were washed with PBS and fixed with 1% paraformaldehyde. Paraffin-embedded mouse tissues were derived from experiments performed by Schneditz and colleagues. In short, antibiotic-induced dysbiosis was simulated in C57BL/6 mice by amoxicillin/clavulanate and indometacin treatment and intragastrical infection with AHC6 or Mut-89, leading to *K. oxytoca* overgrowth in the colon of infected mice [8]. Caecum sections were cut, deparaffinized, and heated in sodium citrate buffer solutions at pH 6.0 for epitope retrieval. After permeabilization with 0.05% Triton X-100, immunostaining of TJ proteins was carried out using anti-ZO-1 or anti-occludin, anti-claudin-1, -5, or -8 (1:100), followed by labelled Alexa Fluor 488 goat anti-mouse or rabbit IgG and Alexa Fluor 594 goat anti-mouse or rabbit IgG (1:1000; Thermo Fisher Scientific). Staining of ZO-1 and claudin-1 on mouse tissue sections was limited to high background staining and weak antibody binding. Therefore, occludin was used in this case. Nuclei were DAPI stained (1:5000). Fluorescence staining was visualized by confocal laser scanning microscopy (LSM 780, Zeiss, Jena, Germany), and tight junction proteins were localized by z-stack imaging. Distribution profiles of claudin signals were generated using Zen software (ZEN 2.3 lite, Zeiss, Oberkochen, Germany).

### 4.7. Statistical Analysis

All values are given as means ± SEM. Data were compared with Student’s t-test and Bonferroni–Holm adjustment in cases of multiple testing. *p* < 0.05 was considered significant (*p < 0.05; **p < 0.01; ***p < 0.001).

## Figures and Tables

**Table 1:** Measurements of permeability to fluorescein (332 Da, P_Fluorescein_) or FITC-dextran 4000 (4 kDa, P_FD4_) and corresponding transepithelial resistance (TER) (mean ± SEM (*n*), * *p* < 0.05; ** *p* < 0.01; *** *p* < 0.001).

	P_Fluorescein_ (10^−6^ cm⋅s^−1^)	P_FD4_ (10^−6^ cm⋅s^−1^)	TER (Ω∙cm^2^)
**TSB**	0.10 ± 0.01 (7)	0.02 ± 0.00 (8)	1754 ± 179 (7)
**AHC6**	1.46 ± 0.31 (7) **	0.09 ± 0.01 (8) ***	231 ± 53 (7) ***
**Mut-89**	0.21 ± 0.02 (6) *	0.03 ± 0.00 (8) *	814 ± 174 (6)

**Table 2:** Densitometric quantification of tight junction protein expression (mean ± SEM (*n*), values are given in % of TSB-treated control, which was set to 100%, * *p* < 0.05; ** *p* < 0.01; *** *p* < 0.001 versus TSB control). QV (Q-VD-OPh).

Tight Junction Protein	Expression (%)				
	AHC6	Mut-89	TSB QV	AHC6 QV	Mut-89 QV
**claudin-1**	65 ± 6 (9) ***	88 ± 11 (9)	81 ± 12 (5)	87 ± 11 (6)	95 ± 11 (6)
**claudin-5**	47 ± 10 (8) **	48 ± 8 (8) ***	90 ± 6 (5)	38 ± 12 (5) *	47 ± 14 (5) *
**claudin-8**	33 ± 5 (9) ***	43 ± 6 (8) ***	102 ± 5 (5)	36 ± 10 (6) **	45 ± 10 (6) **
**claudin-2**	91 ± 11 (6)	78 ± 11 (6)			
**claudin-4**	91 ± 8 (6)	89 ± 9 (6)			
**tricellulin-a**	83 ± 9 (6)	101 ± 7 (6)			

## Abbreviations

AAHC	antibiotic-associated hemorrhagic colitis
FD4	fluorescein-iso-thio-cyanate-dextran-4000
LSM	laser scanning microscopy
*K. oxytoca*	*Klebsiella oxytoca*
QV	Q-VD-Oph
R^epi^	epithelial resistance
R^para^	paracellular resistance
R^trans^	transcellular resistance
TER	transepithelial resistance
TSB	tryptic soy broth
ZO-1	zonula occludens-1
